# Assessment of lixisenatide-induced nephropathy in chick embryos: Implications for prevention of diabetic kidney disease

**DOI:** 10.6026/973206300213926

**Published:** 2025-10-31

**Authors:** Amit Kumar Srivastava, Aishwarya Srivastava, Rohini Srivastava, Swati Yadav, Jyoti Batra, Yogesh Yadav

**Affiliations:** 1 Department of Anatomy, Government Medical College, Datia, Madhya Pradesh, India; 2 Department of Biochemistry, Naraina Medical College and Research Centre, Kanpur, Uttar Pradesh, India; 3 Department Pathology, Naraina Medical College and Research Centre, Kanpur, Uttar Pradesh, India; 4 Department of Anatomy, Santosh Medical College and Hospital, Ghaziabad, Uttar Pradesh, India; 5 Dean Research, Central Research Facility, Santosh Deemed to be University, Ghaziabad, Uttar Pradesh, India; 6 Department of Anatomy, Saraswathi Institute of Medical Sciences, Hapur, Uttar Pradesh, India

**Keywords:** Lixisenatide, diabetic kidney disease, chick embryo, nephroprotection, GLP-1 receptor agonist, oxidative stress

## Abstract

Diabetic kidney disease (DKD) is a major cause of end-stage renal disease and the direct renal effects of GLP-1 receptor agonists remain underexplored. Using a hyperglycemic chick embryo model (n=120), we evaluated dose-dependent nephroprotective effects of lixisenatide. Hyperglycemia induced renal hypertrophy, histological injury, oxidative stress, inflammation and apoptosis. Lixisenatide, particularly at high doses, significantly ameliorated these changes, normalizing kidney-to-body ratios and reducing MDA, TNF-α and caspase-3 activity. Thus, we show that lixisenatide directly preserves renal structure and function independent of systemic metabolic control.

## Background:

Diabetic kidney disease (DKD) is a severe microvascular complication of diabetes mellitus and the foremost cause of chronic kidney disease (CKD) and end-stage renal disease (ESRD) globally [1]. The pathogenesis of DKD is multifactorial, driven primarily by chronic hyperglycemia, which initiates a cascade of detrimental cellular events including glomerular hyperfiltration, renal hypertrophy, oxidative stress, chronic inflammation and ultimately, glomerulosclerosis and tubulointerstitial fibrosis [2, 3]. Despite advances in glycemic control and blockade of the renin-angiotensin-aldosterone system (RAAS), a significant residual risk of DKD progression remains, necessitating the exploration of novel therapeutic strategies. In recent years, glucagon-like peptide-1 receptor agonists (GLP-1 RAs) have emerged as a promising class of anti-diabetic agents with pleiotropic benefits extending beyond glucose regulation. Large-scale cardiovascular outcome trials have consistently demonstrated that GLP-1 RAs, such as liraglutide and semaglutide, significantly reduce the risk of major adverse cardiovascular events and slow the progression of renal complications, primarily by reducing the onset of macroalbuminuria [4, 5]. These nephroprotective effects are partially attributed to systemic improvements in glycemic control, blood pressure and body weight. However, growing preclinical evidence suggests that GLP-1 RAs may also exert direct protective actions on renal cells via GLP-1 receptor activation, which is expressed in various kidney cell types, including glomerular endothelial cells and podocytes [6]. Lixisenatide is a short-acting, prandial GLP-1 RA. The Evaluation of Lixisenatide in Acute Coronary Syndrome (ELIXA) trial showed its cardiovascular safety but did not demonstrate superiority over placebo for renal outcomes, although a trend toward reduced new-onset macroalbuminuria was observed [7]. The limited scope of clinical renal endpoints and the complex interplay of systemic factors in human trials make it challenging to isolate the direct renal effects of lixisenatide. Disentangling these direct actions from indirect, systemic benefits is crucial for understanding their full therapeutic potential in DKD prevention. To address this research gap, an experimental model that allows for the direct assessment of drug effects on kidney development and injury, while minimizing confounding systemic variables, is required. The chick embryo model offers a unique advantage in this context. Its rapid renal (mesonephric and metanephric) development occurs in a closed system, isolated from maternal metabolic influences or pre-existing comorbidities [8]. Inducing hyperglycemia in this model provides a platform to study the direct cellular responses to glucotoxicity and the potential protective effects of therapeutic agents on organogenesis and tissue integrity [9]. Previous studies have successfully used this model to investigate the teratogenic effects of hyperglycemia on various organ systems, but its application to explore nephroprotective pharmacology remains limited. Therefore, it is of interest to utilize a hyperglycemic chick embryo model to investigate the direct, dose-dependent nephroprotective effects of lixisenatide.

## Materials and Methods:

A total of 120 specific pathogen-free (SPF) fertilized White Leghorn chicken eggs were obtained from a commercial hatchery. The eggs were incubated in a digital forced-draft incubator at a constant temperature of 37.8 ± 0.2°C and 60 ± 5% relative humidity, with automatic rotation every hour. On Embryonic Day 2 (E2), eggs were candled to confirm viability. Viable eggs were randomly allocated into six experimental groups (n=20 eggs per group):

[1] Control (CTL): Injected with 100 µL of sterile 0.9% saline.

[2] Hyperglycemia (HG): Injected with 100 µL of 20% D-glucose in sterile saline to induce a hyperglycemic state.

[3] HG + Low-Dose Lixisenatide (HG+Lixi-L): Injected with D-glucose followed by lixisenatide at a dose of 5 nmol/kg egg weight.

[4] HG + Medium-Dose Lixisenatide (HG+Lixi-M): Injected with D-glucose followed by lixisenatide at a dose of 10 nmol/kg egg weight.

[5] HG + High-Dose Lixisenatide (HG+Lixi-H): Injected with D-glucose followed by lixisenatide at a dose of 20 nmol/kg egg weight.

[6] Lixisenatide High only (Lixi-H): Injected with saline followed by lixisenatide at a dose of 20 nmol/kg to assess potential drug toxicity.

## Injection procedure:

Lixisenatide (Sanofi, Paris, France) was reconstituted in sterile saline. On E3, a small hole was drilled through the shell over the air sac under sterile conditions. The HG and HG+Lixisenatide groups were injected with D-glucose solution. The CTL and Lixi-H only groups were injected with saline. One hour later, on E3, the lixisenatide or corresponding saline vehicle injections were administered. The holes were sealed with sterile paraffin wax and eggs were returned to the incubator until E18.

## Sample collection and morphometric analysis:

On E18, embryos were carefully extracted from the eggs and viability was confirmed. Embryos were euthanized by decapitation. The total body weight of each embryo was recorded. The metanephric kidneys were carefully dissected under a stereomicroscope, blotted dry and weighed. The kidney-to-embryo weight ratio (%) was calculated as (total kidney weight / total embryo weight) x 100. For each embryo, one kidney was fixed for histopathology and the other was snap-frozen in liquid nitrogen and stored at -80°C for biochemical analysis.

## Histopathological examination:

Kidney samples were fixed in 10% neutral buffered formalin for 48 hours, processed through a graded series of ethanol, cleared in xylene and embedded in paraffin wax. Sections of 5 µm thickness were cut and stained with Hematoxylin and Eosin (H & E) for general morphology and Masson's trichrome for assessment of collagen deposition and interstitial fibrosis. All slides were examined under a light microscope (Olympus BX53) by an experienced pathologist blinded to the experimental groups. For quantitative analysis, the cross-sectional area of 20 randomly selected glomeruli per kidney section was measured using Image J software (NIH, USA). A semi-quantitative tubular injury score (0-4 scale) was assigned based on the percentage of tubules showing epithelial flattening, vacuolization and dilation (0: <5%, 1: 5-25%, 2: 26-50%, 3: 51-75%, 4: >75%).

## Biochemical assays:

Frozen kidney tissues were homogenized in ice-cold phosphate-buffered saline (PBS) containing a protease inhibitor cocktail. The homogenate was centrifuged at 12,000 x g for 15 minutes at 4°C and the supernatant was collected. Total protein concentration was determined using a Bicinchoninic Acid (BCA) Protein Assay Kit (Thermo Fisher Scientific, USA).

[1] Oxidative stress markers: Lipid peroxidation was quantified by measuring malondialdehyde (MDA) levels using a Thiobarbituric Acid Reactive Substances (TBARS) assay kit (Cayman Chemical, USA). Superoxide dismutase (SOD) activity was measured using a commercial SOD assay kit (Cayman Chemical, USA), which utilizes a tetrazolium salt for the detection of superoxide radicals.

[2] Inflammatory marker: The concentration of tumor necrosis factor-alpha (TNF-α) in the tissue homogenates was measured using a chicken-specific TNF-α ELISA kit (MyBioSource, Inc., USA) according to the manufacturer's protocol.

[3] Apoptosis marker: Caspase-3 activity was measured using a colorimetric assay kit (Abcam, UK), which detects the cleavage of the substrate DEVD-p-nitroanilide. Activity was expressed as a percentage relative to the control group.

## Statistical analysis:

All data are presented as mean ± standard deviation (SD). Statistical analysis was performed using GraphPad Prism version 9.0 (GraphPad Software, USA). Differences between multiple groups were analyzed using a one-way analysis of variance (ANOVA) followed by Tukey's post-hoc test for multiple comparisons. A p-value of < 0.05 was considered statistically significant.

## Results:

As shown in Table 1 (see PDF), embryos in the HG group exhibited a slightly lower, though not statistically significant, total body weight compared to the control group. However, the absolute kidney weight and the kidney-to-embryo weight ratio were significantly increased in the HG group compared to controls (0.58 ± 0.05% vs. 0.45 ± 0.04%, p<0.001), indicating hyperglycemia-induced renal hypertrophy. Treatment with medium (Lixi-M) and high (Lixi-H) doses of lixisenatide significantly and dose-dependently reduced this hypertrophy. The kidney-to-embryo weight ratio in the HG+Lixi-H group (0.47 ± 0.05%) was statistically indistinguishable from the control group. The low-dose (Lixi-L) group showed a minor, non-significant reduction. The Lixi-H only group showed no significant differences in any morphometric parameters compared to the control group. H & E staining of kidney sections from the control group revealed normal renal architecture with well-defined glomeruli and tubules. In contrast, the HG group exhibited marked histopathological abnormalities, including significant glomerular hypertrophy, mesangial expansion and widespread tubular epithelial cell vacuolization and dilation. Masson's trichrome staining showed a noticeable increase in collagen deposition in the tubulointerstitial space in the HG group compared to controls. Quantitative analysis confirmed these observations (Table 2 - see PDF). The mean glomerular area was significantly larger in the HG group (1854 ± 155 µm^2^) compared to the control group (1231 ± 98 µm^2^, p<0.001). The tubular injury score was also significantly elevated in the HG group. Lixisenatide treatment dose-dependently attenuated these changes. The HG+Lixi-H group showed a significant reduction in glomerular area (1315 ± 112 µm^2^, p<0.001 vs. HG) and tubular injury score (1.2 ± 0.4, p<0.001 vs. HG) to levels near those of the control group. To elucidate the mechanisms underlying the protective effects of lixisenatide, we assessed key biochemical markers of renal injury (Table 3 - see PDF). The HG group showed a profound state of oxidative stress, characterized by a more than two-fold increase in the lipid peroxidation product MDA (14.8 ± 1.6 nmol/mg protein) and a concomitant >50% reduction in the activity of the antioxidant enzyme SOD (18.5 ± 2.4 U/mg protein) compared to controls (p<0.001 for both). Lixisenatide treatment dose-dependently reversed these changes. The high dose of lixisenatide almost completely normalized MDA levels and restored SOD activity. Furthermore, hyperglycemia induced a significant inflammatory response, as indicated by a 2.6-fold increase in renal TNF-α levels in the HG group versus controls (p<0.001). Apoptosis was also significantly upregulated, with caspase-3 activity increasing to 215.7 ± 22.1% of the control level (p<0.001). Treatment with medium and high doses of lixisenatide significantly suppressed both TNF-α production and caspase-3 activation in a dose-dependent fashion. The Lixi-H only group exhibited no significant changes in any biochemical parameter compared to the control group, confirming the drug's safety at the tested dose.

## Discussion:

This study provides the first evidence for the direct, dose-dependent nephroprotective effects of lixisenatide in an *in vivo* model of hyperglycemia-induced renal injury. Using the chick embryo, which allows for the isolation of organ-level drug effects from systemic metabolic confounders, we demonstrated that lixisenatide significantly mitigates renal hypertrophy, histopathological damage, oxidative stress, inflammation and apoptosis. These findings suggest that the renal benefits of lixisenatide may extend beyond its well-established role in glycemic control. The induction of hyperglycemia in our model successfully recapitulated key early features of DKD. The observed renal hypertrophy, evidenced by the increased kidney-to-embryo weight ratio and glomerular enlargement, is a classic initial response to hyperglycemia and a predictor of subsequent renal function decline [10]. Lixisenatide's ability to normalize these morphometric changes, particularly at medium and high doses, suggests a direct inhibitory effect on pathological growth pathways within the kidney. This is consistent with studies on other GLP-1 RAs, which have been shown to modulate pro-hypertrophic signaling pathways such as the mammalian target of rapamycin (mTOR) in renal cells [11]. A central finding of our study is the powerful effect of lixisenatide on key pathogenic mechanisms of DKD. Hyperglycemia-induced oxidative stress is a primary driver of renal cell damage [2]. We observed a marked increase in the lipid peroxidation marker MDA and a depletion of the antioxidant enzyme SOD in the hyperglycemic group. Lixisenatide administration dose-dependently reversed this imbalance, indicating a potent antioxidant effect. The activation of the GLP-1 receptor is known to enhance the expression of antioxidant genes through pathways like the nuclear factor erythroid 2-related factor 2 (Nrf2) pathways and to reduce mitochondrial reactive oxygen species (ROS) production [12, 13]. Our results suggest that lixisenatide shares these antioxidant properties, which likely contribute significantly to its renoprotective capacity. Inflammation is another critical component in the progression of DKD. Pro-inflammatory cytokines like TNF-α are upregulated in the diabetic kidney, where they promote endothelial dysfunction, podocyte injury and fibrosis [3]. Our finding that lixisenatide significantly suppressed the hyperglycemia-induced surge in renal TNF-α level aligns with the known anti-inflammatory properties of GLP-1 RAs. This effect is thought to be mediated, in part, by the inhibition of the NF-κB signaling pathway, a master regulator of inflammation [14]. By dampening the inflammatory milieu, lixisenatide may help preserve the structural and functional integrity of the glomeruli and tubules. Finally, our study demonstrated that lixisenatide provides an anti-apoptotic effect. The increased caspase-3 activity in the hyperglycemic kidneys indicates an active process of programmed cell death, which contributes to nephron loss over time. The significant reduction in caspase-3 activity by lixisenatide suggests that it promotes renal cell survival in a glucotoxic environment. This anti-apoptotic action is a recognized feature of GLP-1 receptor activation, often linked to the PI3K/Akt survival signalling pathway [15, 16-17]. A notable aspect of our results is the clear dose-dependency. The low dose of lixisenatide (5 nmol/kg) conferred only marginal protection, whereas the medium (10 nmol/kg) and high (20 nmol/kg) doses showed robust and progressively stronger effects across all measured parameters. This highlights the importance of achieving adequate therapeutic concentrations to elicit direct renal benefits and may have implications for clinical dosing strategies aimed at organ protection. Several limitations of this study must be acknowledged. The chick embryo is an avian, not a mammalian, model. While it is excellent for studying developmental toxicity and direct drug effects, differences in renal physiology exist. Furthermore, the acute hyperglycemic insult induced in this model does not replicate the chronic, progressive nature of DKD in humans, which involves advanced fibrosis and a decline in glomerular filtration rate. Therefore, the long-term effects of lixisenatide on these advanced stages of DKD cannot be inferred from our data. Future studies in chronic mammalian models of DKD are warranted to confirm these findings and to explore the impact of lixisenatide on functional parameters like albuminuria and GFR.

## Conclusion:

Lixisenatide confers direct and dose-dependent nephroprotection in a hyperglycemic chick embryo model is shown. These protective effects are mediated through the concurrent mitigation of renal hypertrophy, oxidative stress, inflammation and apoptosis. The findings strongly suggest that lixisenatide's renoprotective potential is not merely a by-product of its glucose-lowering action but involves direct engagement with beneficial cellular pathways within the kidney. This work provides a compelling mechanistic rationale for the use of lixisenatide in the early prevention of diabetic kidney disease and supports further investigation into the role of GLP-1 RAs as primary renal-protective agents in patients with diabetes.
